# Coracoid Process Avulsion Fracture at the Coracoclavicular Ligament Attachment Site in an Osteoporotic Patient with Acromioclavicular Joint Dislocation

**DOI:** 10.1155/2016/9580485

**Published:** 2016-07-17

**Authors:** Yoshihiro Onada, Takahisa Umemoto, Kimitaka Fukuda, Tomomichi Kajino

**Affiliations:** ^1^Department of Orthopaedic Surgery, Tonan Hospital, Kita-1-jou Nishi-6-chome, Chuo-ku, Sapporo, Hokkaido 060-0001, Japan; ^2^Itou Orthopaedic Hospital, Minami-2-jou Nishi-10-chome 5, Chuo-ku, Sapporo, Hokkaido 060-0062, Japan

## Abstract

Coracoid fractures are uncommon, mostly occur at the base or neck of the coracoid process (CP), and typically present with ipsilateral acromioclavicular joint (ACJ) dislocation. However, CP avulsion fractures at the coracoclavicular ligament (CCL) attachment with ACJ dislocation have not been previously reported. A 59-year-old woman receiving glucocorticoid treatment fell from bed and complained of pain in her shoulder. Radiographs revealed an ACJ dislocation with a distal clavicle fracture. Three-dimensional computed tomography (3D-CT) reconstruction showed a small bone fragment at the medial apex of the CP. She was treated conservatively and achieved a satisfactory outcome. CP avulsion fractures at the CCL attachment can occur in osteoporotic patients with ACJ dislocations. Three-dimensional computed tomography is useful for identifying this fracture type. CP avulsion fractures should be suspected in patients with ACJ dislocations and risk factors for osteoporosis or osteopenia.

## 1. Introduction

Coracoid fractures with ipsilateral acromioclavicular joint (ACJ) dislocation usually occur at the base or neck of the coracoid process (CP), with an intact coracoclavicular ligament (CCL) attached to the fracture fragment [1–4]. However, CP avulsion fractures at the CCL attachment have not been previously reported in patients with ACJ dislocations [5–7]. We report the case of a woman with a CP avulsion fracture after high-dose glucocorticoid (GC) treatment.

## 2. Case Report

**Figure 1:**
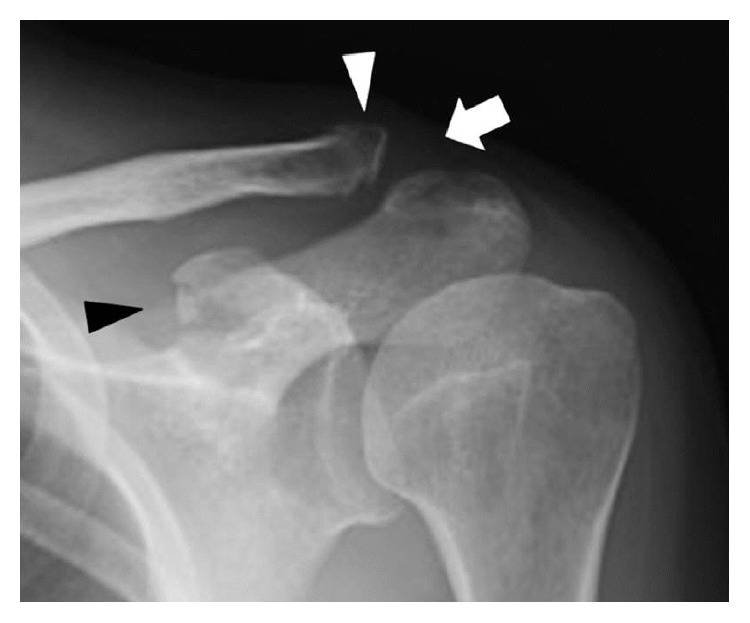
Radiograph showing an ACJ dislocation (white arrow) with distal clavicle fragment displaced upwards (white arrowhead). Upon close examination, a cortical bone discontinuity at the CP medial apex is observed (black arrowhead).

**Figure 2:**
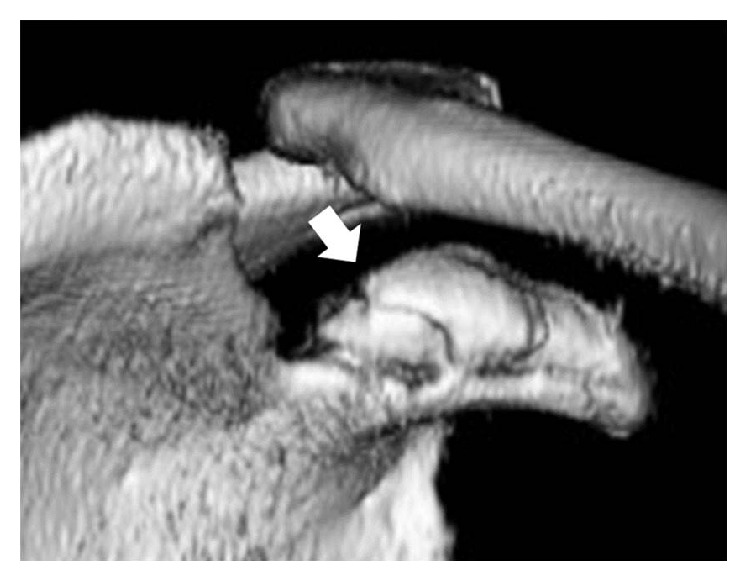
A 3D-CT reconstruction clearly reveals a small bone fragment at the CP medial apex (arrow).

A 59-year-old woman with follicular lymphoma was initially treated with six cycles of chemotherapy and prednisolone. After 2 months, the patient had received a total of 1350 mg of prednisolone and was on her fourth cycle of chemotherapy. She fell from bed and sustained a blow to her left shoulder. She complained of pain in her left shoulder and was examined on the same day. The shoulder was swollen with tenderness over the ACJ. Radiographs showed an ACJ dislocation with a distal clavicle fracture and CP deformity (Figure 1). Three-dimensional computed tomography (3D-CT) reconstruction clearly showed a small CP bone fragment at the CCL insertion site (Figure 2). We diagnosed the patient with a CP avulsion fracture. She was treated nonoperatively in a sling for 3 weeks, followed by progressive range-of-motion exercises. One month after her injury, the patient's shoulder functioned normally without pain.

## 3. Discussion

This case demonstrates that CP avulsion fractures at the CCL attachment can occur in osteoporotic patients with ACJ dislocations and that 3D-CT is useful for identifying these injuries. CP avulsion fractures at the CCL attachment site may be a consequence of GC-induced bone fragility, as GC treatment is a strong risk factor for osteoporosis and fractures.

GC is associated with bone mineral density loss and bone quality deterioration [8–11]. GC treatment is associated with a rapid increase in fracture risk within the first 3 months of treatment [8]. GC-induced fracture risk is also related to the patient's daily GC dose [12]. Avulsion injuries of the coracoid epiphyseal plate near the CCL attachment often occur in younger patients with ACJ dislocations [13, 14]. A shell-like ossification center over the tip of the CP is present until epiphyseal fusion at 18–25 years of age [15]. However, in patients with closed physes, complete ACJ dislocations usually involve CCL disruption because the CP and clavicle are stronger than the ligament [14]. A CP avulsion fracture may have occurred in this 59-year-old woman because GC-induced osteoporotic bone is weaker than the ligament. To our knowledge, this is the first report of a CP avulsion fracture in a patient with closed physes.

CP fracture treatment is based on the location and displacement. The presence of associated injuries is also considered. However, CP fractures are difficult to visualize on standard radiographs because of the marked foreshortening and projection of the acromion or scapular blade [3]. In a combined injury, CP fractures are easily overlooked as the more obvious ACJ dislocation is diagnosed [3, 4]. Therefore, special radiographic views or CT scans are needed [1, 4]. Here, 3D-CT revealed a small bone fragment on the posteromedial CP. The CCL is composed of the conoid and trapezoid ligaments. The conoid ligament arises from the CP posterior and medial to the attachment of the trapezoid ligament [2]. In this case, a pure CP avulsion fracture occurred at the attachment of the conoid ligament.

We must be aware that osteoporotic patients presenting with ACJ dislocations may also have CP avulsion fractures. There may be many more cases of overlooked CP avulsion fractures because of the small bone fragment size and the more obvious ACJ dislocation. In patients at risk for osteoporosis or osteopenia after ACJ dislocation, CP avulsion fractures should be suspected. Three-dimensional CTs are helpful for diagnosing the avulsed bone fragment if the CP appears abnormal on radiographs. Further reports are needed to determine whether CP avulsion fractures may be more common than previously thought.
